# Effect of over expressing protective antigen on global gene transcription in *Bacillus anthracis* BH500

**DOI:** 10.1038/s41598-018-34196-y

**Published:** 2018-10-31

**Authors:** Ashish K. Sharma, Stephen H. Leppla, Andrei P. Pomerantsev, Joseph Shiloach

**Affiliations:** 1 Biotechnology Core Laboratory, National Institute of Diabetes and Digestives and Kidney Diseases (NIDDK) NIH, Maryland, USA; 2 Microbial Pathogenesis Section, National Institute of Allergy and Infectious diseases (NIAID), NIH, Maryland, USA

## Abstract

Protective antigen (PA) of *Bacillus anthracis* is being considered as a vaccine candidate against anthrax and its production has been explored in several heterologous host systems. Since the systems tested introduced adverse issues such as inclusion body formation and endotoxin contamination, the production from *B. anthracis* is considered as a preferred method. The present study examines the effect of PA expression on the metabolism of *B. anthraci*s producing strain, BH500, by comparing it with a control strain carrying an empty plasmid. The strains were grown in a bioreactor and RNA-seq analysis of the producing and non-producing strain was conducted. Among the observed differences, the strain expressing rPA had increased transcription of *sigL*, the gene encoding RNA polymerase σ^54^, *sigB*, the general stress transcription factor gene and its regulators *rsbW and rsbV*, as well as the global regulatory repressor *ctsR*. There were also decreased expression of intracellular heat stress related genes such as *groL, groES, hslO, dnaJ*, and *dnaK* and increased expression of extracellular chaperons *csaA* and *prsA2*. Also, major central metabolism genes belonging to TCA, glycolysis, PPP, and amino acids biosynthesis were up-regulated in the PA-producing strain during the lag phase and down-regulated in the log and late-log phases, which was associated with decreased specific growth rates. The information obtained from this study may guide genetic modification of *B. anthracis* to improve PA production.

## Introduction

Protective antigen (PA) is 83-kDa protein, a component of anthrax exotoxin, which in addition to PA contains the lethal factor (LF) and edema factor (EF) proteins. The complete toxin is an example of the A-B toxin superfamily. PA generates a strong antibody response that is protective against anthrax infection^1^ and therefore is the preferred choice for vaccine development. Initially PA was produced in *Bacillus anthracis*^2,3^, but since this organism is associated with several handling restrictions together with relatively low production^2^, production of a recombinant PA has been explored in various bacterial hosts such as *E. coli, Bacillus subtilis, and Baculovirus*. However, protein production from these host systems can be associated with low productivity, inclusion body formation, contamination from lipopolysaccharide, etc.^4–10^. Therefore, expressing this protein in a modified, nonpathogenic, *B. anthracis* host may offer an attractive strategy. Here we employed the *B. anthracis* strain BH500, which is asporogenic, lacks both virulence plasmids, and is deleted for ten extracellular proteases^11^.

A logical approach to improving a production host is to identify limiting/restricting nodes/pathways and then altering their expression accordingly. Gene expression proofing tools like microarray^12–16^ and RNA-seq^17^ have been successfully applied to different microbial cell factories for identification of plausible bottlenecks that limit expression of a desired recombinant protein. Huang *et al*.^17^ showed that secretion of α-amylase from yeast was affected due to attuned energy metabolism, amino acid biosynthesis, and thiamine biosynthesis. The findings were used to inverse engineer the strain to improve secretion efficiency^17^. Similar work in *Pichia pastoris* identified the ER trafficking gene WSC4 and the ergosterol pathway as bottlenecks; their modification lead to a 2-fold increase in Fab production^12^. In a study of production of an insulin-like growth factor I fusion protein (IGF-I_f_) in *E. coli*, Choi *et al*. found down regulation of *prsA*, a gene required for *E. coli* nucleotide and amino acid biosynthesis. Its over expression improved production from 1.8 to 4.3 g/L in high cell density cultures^18^. Transcriptomic profiling also showed that bottlenecks can develop in different pathways in *E. coli* depending on the type and behavior of the recombinant protein, e.g. interferon-β (inclusion body), xylanase (soluble), and GFP (soluble)^16^. Marciniak *et al*. showed that by using 8 different recombinant proteins that *B. subtilis* gene responses depended on both the origin of the proteins (endogenous vs. heterologous) and on their cellular localization (secreted, membrane, lipid anchored)^19^. At the present time, there is no information on gene expression patterns in *B. anthracis* expressing recombinant proteins at high cell density. Most transcriptome studies in *B. anthracis* have focused on networks involved in host-pathogen interactions^20,21^ and metabolism^22^.

The present study seeks to identify plausible bottlenecks restricting overexpression of PA protein by analyzing the whole genome transcriptional changes in producing and non-producing (control) recombinant BH500 strains grown in a bioreactor. Preliminary studies showed that genes present in the backbone of the empty pSW4 vector cause a significant decrease in the growth rate when compared to the untransformed BH500 strain. Therefore, to identify transcriptional changes caused specifically by PA expression, we compared strains containing either the *pagA* gene-containing pYS5 plasmid or the empty parental vector pSW4. Changes in gene expression were determined for bioreactor-grown cultures sampled in lag, log, and late-log phases. The differences seen in essential pathways required for protein expression including: central carbon metabolism, amino acid biosynthesis, transcription, translation, folding and secretion, were evaluated to identify plausible bottlenecks. The genes identified provide targets for genetic engineering to increase the effectiveness of *B. anthracis* strains as production hosts^11^.

## Results

### Growth of *B. anthracis* BH500 expressing and not expressing recombinant protective antigen (rPA)

**Figure 1:**
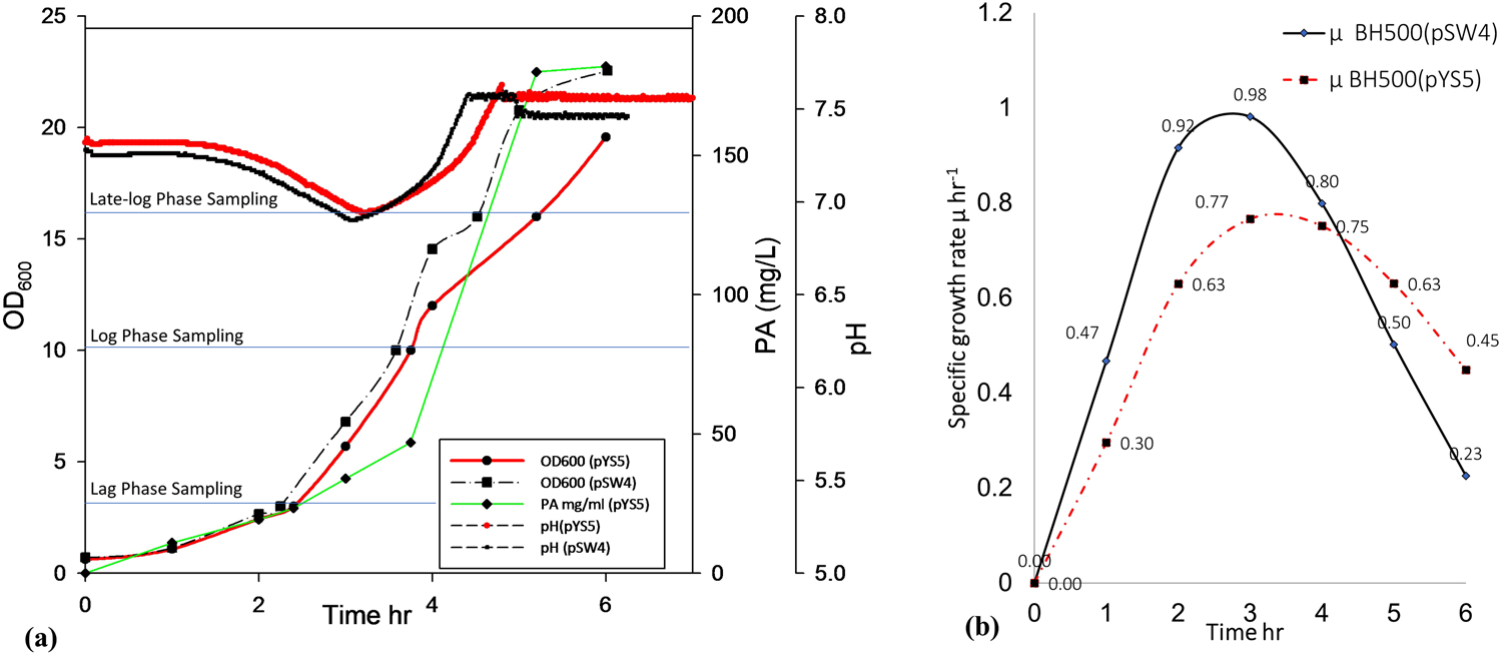
(**a**) Growth and production pattern of *Bacillus anthracis* expressing PA (pYS5) and the control strain carry plasmid without PA (pSW4); (**b**) Specific growth rate profile of the expressing (pYS5) and the non-expressing (pSW4) cultures.

Growth parameters of two *B. anthracis* BH500 strains, one harboring plasmid pYS5 expressing PA and the other harboring control plasmid pSW4, are shown in Fig. 1(a). PA expressing and non-expressing cultures were grown without kanamycin selection pressure, where plasmid stability tests confirmed the culture purity and no generation of non-recombinants. Kanamycin was avoided since previous studies showed a decrease in the growth rate of cultures growing in the presence of kanamycin compared with cultures without kanamycin. The specific growth rates of the two cultures are seen in Fig. 1(b). The strain expressing PA reached a maximum of 0.8 h^−1^ and then declined as the culture OD_600_ exceeded 10, whereas the control strain reached a maximum specific growth rate of 1 h^−1^ that started to decline as the culture reached OD_600_ ~ 6. The highest PA expression at the end of the batch run was ~180 mg/L. The lag, log, and late-log growth phase samples of PA expressing, and non-expressing culture were processed with live/dead cell assay, which showed no significant difference and thus PA expression had no significant effect on cell viability.

### Transcriptome analysis of the PA-producing and non-producing strains during lag, log, and late-log phases

**Figure 2:**
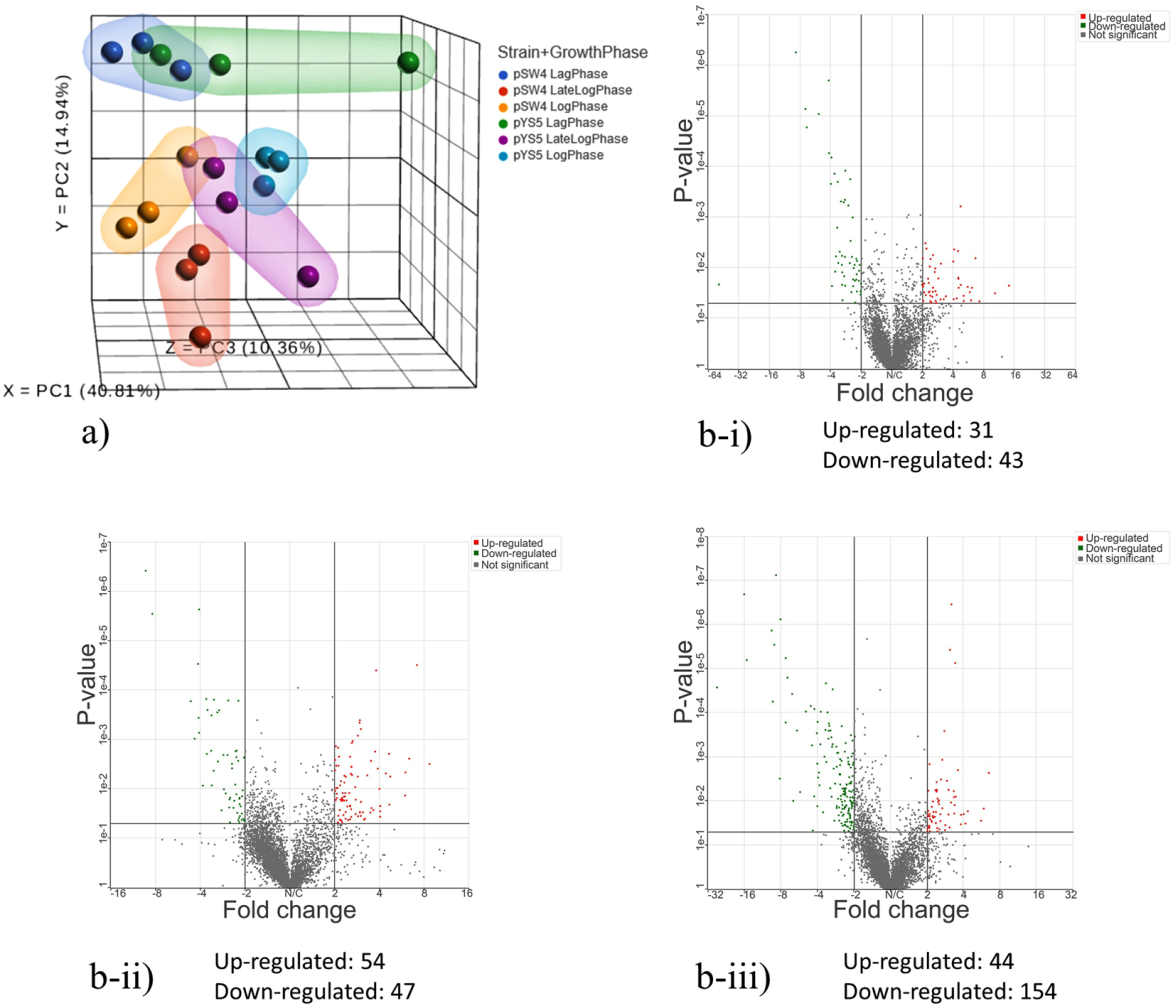
(**a**) Principal component analysis (PCA) of triplicates transcriptome data of the 2 stains at three growth phases; (**b**) Volcano plot representing fold change distribution of gene expressions at i) lag, ii) log and iii) late-log phases between PA producing (pYS5) and non-producing (pSW4) strains. Red and green dots representing up and down-regulated genes respectively.

Gene transcription analyses of the strain producing recombinant PA and the control strain at lag, log, and late-log growth phases were performed by quantifying transcript levels using RNA-seq (Table 1). RNA samples from lag, log, and late-log phase cultures were converted to cDNA and sequenced on the Illumina Hiseq 2500 platform. Triplicate data were obtained from the biological replicates of the three growth phases of PA expressing and non-expressing cultures. Average read quality was close to ~40% in all samples and fraction of no calls (%N) was 0% in all samples. The single end sequencing was done for 50 base pair amplifications and the total number of reads for each sample was above the acceptable range. The unaligned reads were then trimmed to remove the low-quality bases, identified by the probability that they are called incorrectly. All samples were ~97% uniquely aligned with the reference genome and seem to be of satisfactory quality. Principal component analysis (PCA) showed that the principal gene components in the biological triplicates of RNAseq data were distributed close to each other, representing a higher correlation (Fig. 2(a)). PCA also represented a significant difference in the overall gene expression distribution among the biological triplicate sets of samples from PA expressing and non-expression cultures. By aligning transcripts with the reference *B. anthracis* Ames ancestor genome (AE017334.2), transcripts for 5507 genes were identified. Statistical analysis using ANOVA was applied on the 5507 genes, which identified 3212 differentially expressed genes. By applying further filtering criteria of p value ≤ 0.05 and fold change (FC) cut-off <2>, 31 up-regulated and 43 down-regulated genes were identified in the lag phase of the PA-producing culture as compared to the control. The analysis identified 54 up-regulated and 47 down-regulated genes in log phase, 44 up-regulated, and 154 down-regulated genes in the late-log phase (Fig. 2(b)).

**Table 1:** Summary of RNA-sequencing reads.

Batch Run	StrainType	OD600	Total reads (X 106)	Total unique (X 106)	Unique	Total alignments (X 106)	Aligned	Coverage	Avg. coverage depth
1	pSW4	3	17.77	17.28	0.97	17.35	0.97	0.62	271
1	pSW4	10	17.83	17.31	0.97	17.38	0.97	0.64	267
1	pSW4	16	18.69	18.26	0.98	18.33	0.98	0.58	309
2	pSW4	3	20.97	20.38	0.97	20.47	0.97	0.66	303
2	pSW4	10	18.39	17.78	0.97	17.85	0.97	0.72	243
2	pSW4	16	16.91	16.46	0.97	16.52	0.98	0.62	259
3	pSW4	3	15.48	14.96	0.97	15.01	0.97	0.68	217
3	pSW4	10	28.95	27.79	0.96	27.97	0.96	0.77	354
3	pSW4	16	17.24	16.59	0.96	16.66	0.96	0.69	236
4	pYS5	3	26.93	26.18	0.97	26.34	0.98	0.75	340
4	pYS5	10	20.02	19.47	0.97	19.56	0.97	0.65	292
4	pYS5	16	12.75	12.50	0.98	12.54	0.98	0.50	245
5	pYS5	3	20.46	20.07	0.98	20.17	0.98	0.65	304
5	pYS5	10	17.72	17.20	0.97	17.27	0.97	0.67	252
5	pYS5	16	14.21	13.71	0.96	13.75	0.97	0.67	201
6	pYS5	3	25.60	25.05	0.98	25.20	0.98	0.70	352
6	pYS5	10	17.74	17.19	0.97	17.26	0.97	0.71	238
6	pYS5	16	15.26	14.92	0.98	14.98	0.98	0.57	257

A small fraction (2.5–3%) of the transcripts did not align with the *B. anthracis* reference genome. Many of these transcripts aligned with the plasmid sequence and specifically with the *neo, repB* and *bla* genes. As expected, *pagA* transcripts (from rPA gene) were found only in the PA-producing culture. There was 1.36-fold increase in the number of *pagA* transcripts in the PA-producing culture from lag phase to log phase, which then decreased as the culture progressed to late-log phase.

### Transcriptional changes specific to lag, log, or late-log phases

**Figure 3:**
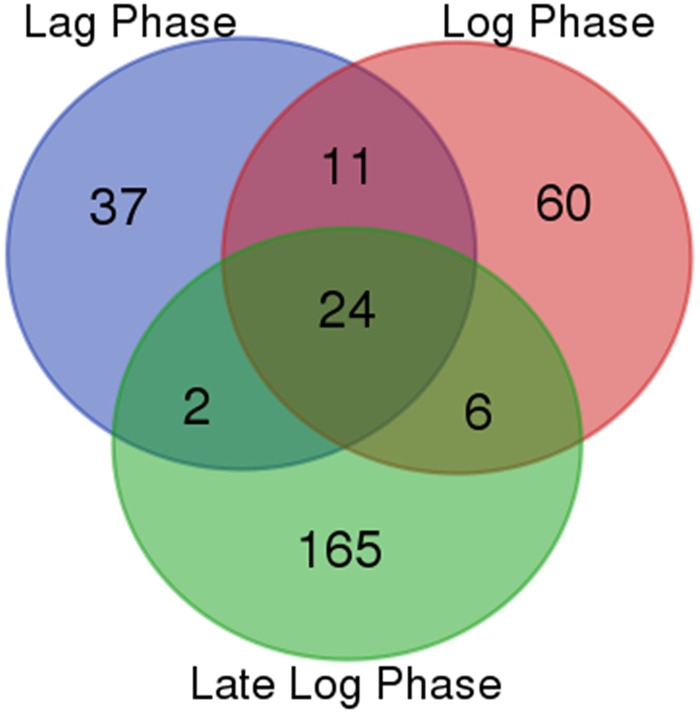
Venn analysis representing the genes commonly up/down-regulated between the 3 growth phases. Genes with list of interest mainly included *Lag Phase Vs Log Phase Vs Late-Log Phase* i.e. list of genes DE during lag, log, and late-log phase (24); *Lag Phase Vs Log Phase* i.e. list of genes commonly DE in lag and late-log phases (11); *Log Phase Vs Late-Log Phase* i.e. list of genes DE during transition from log to late-log phase (6) and *Lag Phase Vs Late-Log Phase* i.e. list of genes DE in lag phase and later became significant in late-log phase (2).

To characterize the distribution of genes expressed differentially in the three growth phases in the producing vs. the non-producing strains, Venn analysis was conducted, producing the result shown in Fig. 3. Twenty-four genes were differentially expressed in all three growth phases. Thirty-seven genes were differentially expressed only in the lag phase and 11 genes were shared between the lag and log phases. Only 2 genes were differentially expressed in the lag and the late-log phases, which clearly shows the distinct differences in gene transcription during the different phases. The log phase had a higher number of differentially expressed genes; there were 60 genes uniquely present in this set. The log phase gene set shared 11 and 6 genes with the lag and late-log phase sets, respectively. The late-log phase gene set was the largest of the three comparison sets where 165 genes expressed differentially, while just 2 and 6 differentially expressed genes were shared with the lag and log phases, respectively. The specific genes in each set, along with their log_2_ fold change expression values, up and down-regulation state and their protein product are summerized in Table 2.

**Table 2:** List of common genes across different phases of batch run in PA expressing and non-expressing control culture. p-value and fold change (log_2_) values are from a comparison of pYS5 LagPhase vs. pSW4 LagPhase, vice versa for log phase and late-log phase.

Count No.	Gene name or identifier	Putative Function	Lag phase			Log phase			Late-log phase		
			P-value	Fold change	Status	P-value	Fold change	Status	P-value	Fold change	Status
***differentially regulated in all growth phases (24)***											
1	pyrE	orotate phosphoribosyltransferase	0.0058	−3.09	Down	0.0007	−4.07	Down	0.0013	−3.38	Down
2	GBAA_3859	heavy metal-transporting ATPase	0.0005	−2.90	Down	0.0002	−3.23	Down	0.0006	−2.79	Down
3	uraA	uracil permease	0.0000	−4.17	Down	0.0000	−4.07	Down	0.0000	−2.99	Down
4	pyrF	orotidine 5′-phosphate decarboxylase	0.0016	−3.45	Down	0.0004	−4.10	Down	0.0003	−4.05	Down
5	pyrD	dihydroorotate oxidase	0.0146	−2.94	Down	0.0019	−3.61	Down	0.0122	−2.49	Down
6	GBAA_0239	hypothetical protein	0.0000	−5.22	Down	0.0008	2.86	Up	0.0012	−2.53	Down
7	GBAA_3492	ABC transporter, efflux permease protein	0.0000	−7.08	Down	0.0000	−8.38	Down	0.0000	−7.30	Down
8	GBAA_3495	conserved hypothetical protein	0.0001	−3.66	Down	0.0003	−3.07	Down	0.0011	−2.60	Down
9	GBAA_4720	thiJ/pfpI family protein	0.0001	−4.14	Down	0.0002	−3.64	Down	0.0004	−3.25	Down
10	GBAA_2880	conserved hypothetical protein	0.0083	3.03	Up	0.0019	4.64	Up	0.0082	3.33	Up
11	GBAA_4963	conserved hypothetical protein	0.0087	−3.11	Down	0.0162	−2.54	Down	0.0139	−2.65	Down
12	pyrC	dihydroorotase	0.0101	−2.38	Down	0.0003	−3.36	Down	0.0051	−2.41	Down
13	GBAA_2879	conserved hypothetical protein	0.0047	4.46	Up	0.0024	6.33	Up	0.0198	4.01	Up
14	nadA	quinolinate synthetase complex, subunit A	0.0002	−2.56	Down	0.0002	−2.59	Down	0.0010	−2.09	Down
15	GBAA_5564	conserved hypothetical protein	0.0296	−2.03	Down	0.0141	−2.10	Down	0.0042	−2.59	Down
16	ctsR	transcriptional regulator CtsR	0.0364	3.39	Up	0.0099	4.00	Up	0.0171	4.33	Up
17	GBAA_3494	conserved hypothetical protein	0.0001	−3.92	Down	0.0003	−2.99	Down	0.0002	−3.19	Down
18	GBAA_1942	bacterial luciferase family protein	0.0080	−3.37	Down	0.0010	−4.36	Down	0.0014	−4.02	Down
19	GBAA_0238	hypothetical protein	0.0002	−3.97	Down	0.0176	2.23	Up	0.0062	−2.37	Down
20	GBAA_4962	transcriptional regulator, ArsR family	0.0048	−3.26	Down	0.0101	−2.77	Down	0.0040	−2.86	Down
21	katB	catalase	0.0119	−3.60	Down	0.0474	−2.52	Down	0.0201	−2.44	Down
22	dra	deoxyribose-phosphate aldolase	0.0029	−2.55	Down	0.0021	−2.79	Down	0.0002	−3.99	Down
23	GBAA_1898	putative membrane protein	0.0000	−6.86	Down	0.0002	−4.62	Down	0.0001	−4.98	Down
***differentially regulated only in Lag and Log Phase (11)***											
1	GBAA_1669	putative flagellar hook-associated protein	0.0247	2.54	Up	0.0121	2.50	Up	0.8290	1.14	Up
2	GBAA_2390	putative ABC transporter, permease protein	0.0302	2.30	Up	0.0453	2.24	Up	0.6881	1.10	Up
3	GBAA_5712	yycI protein	0.0162	2.86	Up	0.0039	3.90	Up	0.0673	2.68	Up
4	GBAA_2878	conserved hypothetical protein	0.0198	2.30	Up	0.0018	3.75	Up	0.0805	2.08	Up
5	GBAA_2929	putative flavoprotein	0.0417	3.23	Up	0.0295	3.10	Up	0.2148	2.08	Up
6	pckA	phosphoenolpyruvate carboxykinase (ATP)	0.0088	−2.08	Down	0.0031	−2.45	Down	0.0798	−1.53	Down
7	GBAA_2458	conserved hypothetical protein	0.0237	5.55	Up	0.0031	8.69	Up	0.1008	4.05	Up
8	GBAA_5260	major facilitator family transporter	0.0061	−2.90	Down	0.0123	−2.69	Down	0.0555	−1.98	Down
9	GBAA_4668	ABC transporter, permease protein	0.0248	6.12	Up	0.0416	3.15	Up	0.3388	1.50	Up
10	GBAA_5510	techoic acid ABC transporter, ATP-binding protein	0.0420	3.00	Up	0.0423	2.47	Up	0.4320	1.57	Up
11	GBAA_0032	conserved hypothetical protein	0.0354	2.39	Up	0.0491	2.24	Up	0.1875	2.03	Up
***differentially regulated only in Lag and Late-log Phase (2)***											
1	GBAA_2140	conserved domain protein	0.0061	−2.47	Down	0.3184	−1.35	Down	0.0043	−2.30	Down
2	GBAA_4669	ABC transporter, ATP-binding protein	0.0416	4.04	Up	0.0752	2.39	Up	0.0410	2.42	Up
***differentially regulated only in Log and Late-log Phase (6)***											
1	GBAA_1162	alpha-amylase family protein	0.3857	1.39	Up	0.0164	2.19	Up	0.0255	2.11	Up
2	hemH2	ferrochelatase	0.0624	−2.01	Down	0.0173	−2.36	Down	0.0069	−2.41	Down
3	carB	carbamoyl-phosphate synthase, large subunit	0.0933	−2.03	Down	0.0017	−3.38	Down	0.0076	−2.57	Down
4	GBAA_2459	hypothetical protein	0.0690	1.74	Up	0.0057	2.38	Up	0.0414	2.02	Up
5	sigB	RNA polymerase sigma-B factor	0.0455	1.72	Up	0.0070	2.21	Up	0.0220	2.04	Up
6	GBAA_4072	conserved domain protein	0.0601	2.92	Up	0.0048	4.42	Up	0.0337	3.80	Up

### Over-representation of differentially expressed genes in various metabolic pathways

**Figure 4:**
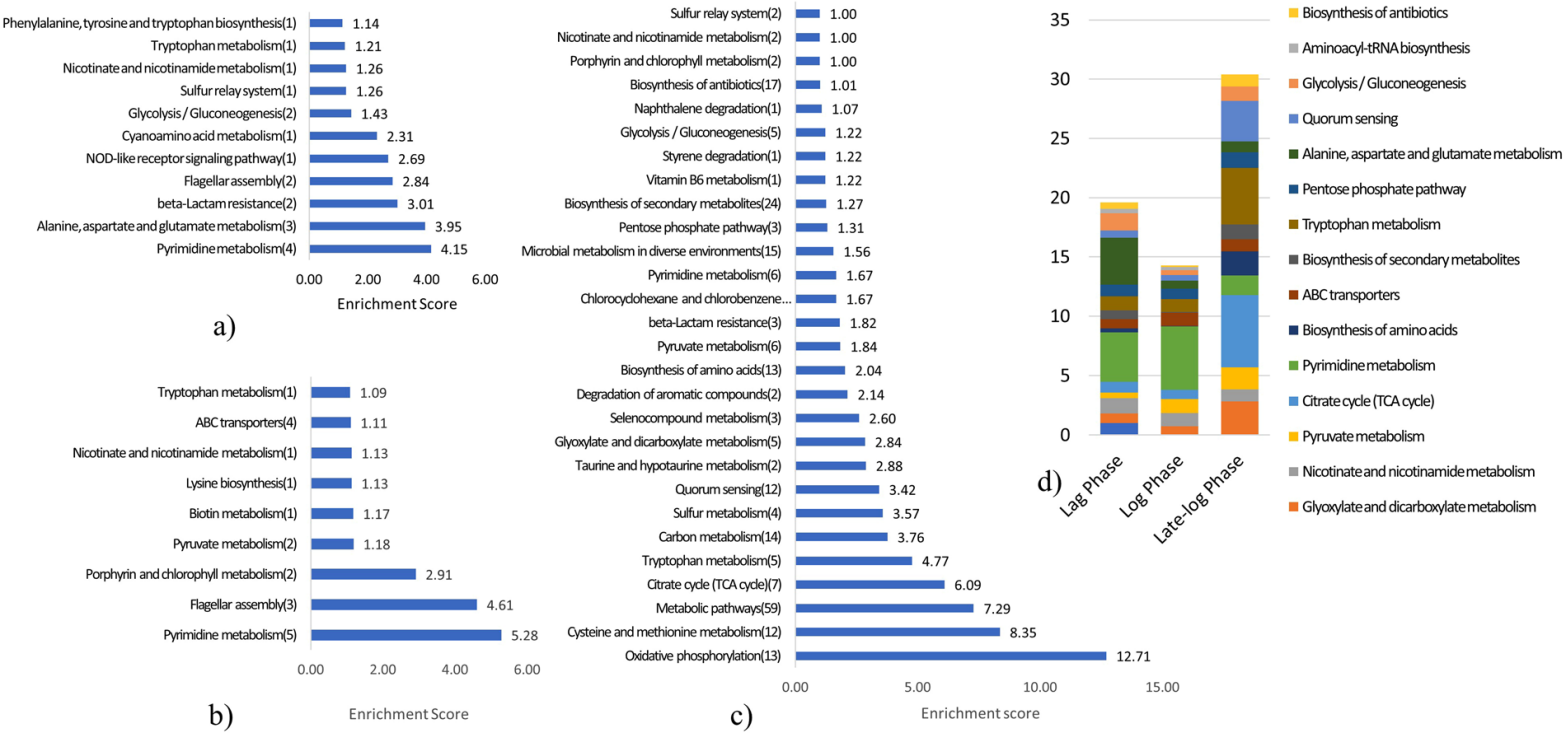
Pathway enrichment analysis of DE gene list from (**a**) lag phase, (**b**) log phase, (**c**) late-log phase, and (**d**) Change in enrichment scores of pathways in lag, log, and late-log phase. (**a**–**c**) Number of over-represented genes is mentioned in front of the pathway name, along with the enrichment score on top of each bar. (**d**)- same number of pathways in each bar showing enrichment scores of pathways as their relative thickness on Y-axis).

Pathway enrichment analysis on the differentially expressed genes from each growth phase was done to identify the effects of PA expression. Bar graph presentation of the important pathways containing differentially expressed genes is shown in Fig. 4, along with the enrichment scores (only pathways with scores >1 are shown). As the cultures progressed from lag to log and late-log, more pathways showed differentially expressed genes. Pyrimidine metabolism was among those most affected, since genes from this class (such as *pyrC, pyrD, pyrE* and *pyrF*) had high enrichment scores in the lag phase. These genes remained overrepresented in log as well as late-log phases together with the *carB*, which encodes a subunit of carbamoyl phosphate synthase and GBAA_3162 (5′-nucleotidase) respectively. Amino acid metabolism which is critical for cell growth was found to be up-regulated in the lag phase, likely to support increased requirements for amino acid for PA biosynthesis. Specifically, alanine, aspartate and glutamate metabolism, tryptophan metabolism, and phenylalanine, tyrosine and tryptophan biosynthesis genes, were overrepresented in the lag phase data. The down-regulation of the amino acid biosynthesis genes in log and late-log phase probably slowed down the overall amino acid metabolism. Lag phase data was mostly overrepresented with energy and central carbon metabolism pathways such as oxidative phosphorylation, glycolysis, and TCA. It is important to mention that in the brief period when the culture progressed from log phase to late-log phase, the number of pathways with enrichment score >1 increased more than three times: 198 genes were differentially expressed in the late-log phase (the largest number of genes compared with the other growth phases). The outcome was enrichment scores >1 in 28 different metabolic pathways (Fig. 4(c)) (The full data set underlying Fig. 4(a–c) is provided in Supplementary Table 1). Late-log phase data showed that 13 genes associated with oxidative phosphorylation (*ctaD, ctaE, nuoD, nuoH, nuoI, nuoJ, nuoK, nuoL, nuoM, nuoN, qcrA, qcrB, sdhC*) were differentially expressed, giving this pathway the highest enrichment score. This group contains genes involved in the biosynthesis of ATP, suggesting that late-log growth imposes a requirement for additional energy generation, inducing genes such as *ctaD* and *ctaE* for cytochrome c oxidase subunit I & III; and *nuoD, nuoH, nuoI, nuoJ, nuoK, nuoL, nuoM*, and *nuoN* encoding different subunits of NADH dehydrogenase; *qseA* and *qseB* coding menquinol-cytochrome c reductase iron sulfur subunit, and *sdhC* of succinate dehydrogenase. In addition, central carbon metabolism pathways were also overrepresented in the late-log phase. This included *fbp2, GBAA_2222, GBAA_4896, pdhA, and pdhB* genes from glycolysis pathways, *acnA, GBAA_1862, pdhA, pdhB, pyc, sdhC, and sucC* from TCA cycle, and *dra, fbp2, and tkt1* from the pentose phosphate pathway. Quorum sensing, sulfur metabolism, and cysteine and methionine metabolism genes were also differentially expressed in late-log phase (Supplementary Table 1).

## Discussion

As shown in the results section, the cells expressing PA grew more slowly than the control cells. This could be due to the diversion of resources to protein expression and/or interference with cell surface processes by high protein secretion. These stresses are expected to lead to changes in gene transcription. The changes identified by RNA-seq in pathways that play vital roles in cellular physiology are summarized here.

### Glycolysis, TCA, pentose phosphate pathway and oxidative phosphorylation

**Figure 5:**
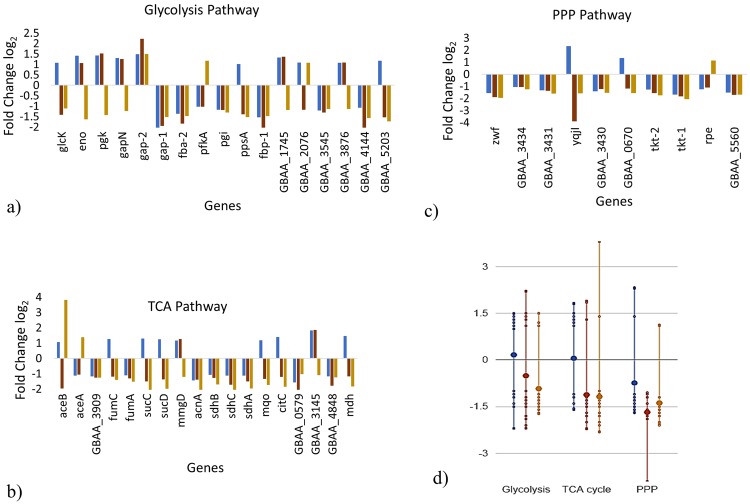
log_2_ fold change values of genes in the energy metabolism pathways (**a**) Glycolysis, (**b**) TCA, (**c**) PPP, (**d**) Overall gene expression distribution in Glycolysis, TCA and PPP. The large dot represents the average (mean) of all data values for subsystem genes belonging to a pathway while small dots represent a data value for an individual subsystem/gene) (generated using EcoCyc omics dashboard). Blue color represent lag phase (pYS5) vs lag phase (pSW4), orange color represent log phase (pYS5) vs log phase (pSW4) and gold color represents late-log phase (pYS5) vs late-log phase (pSW4).

Because of down regulation of *pgi* (−1.17 in lag, −1.19 in log and −1.31 in late-log phases (log_2_ scale)) in the PA producing strain, the expression of the upper glycolytic pathway genes was lower compared with the non-producing strain. At the same time *gap2* encoding glyceraldehyde-3-phosphate dehydrogenase was up-regulated throughout the lag (1.49), log (2.22) and late log phase (1.51), generating NADPH during conversion of 3-phospho-D-glyceroyl phosphate from D-glyceraldehyde 3-phosphate and therefore likely maintained the energy balance of the cell. The upregulation of TCA cycle genes i.e. *fumC, mdh, mqo, citZ, citC, adhB, sucC*, and *sucD* very likely supporting the increased energy requirement in the lag phase by generatng NADH and ATP. However, these same genes were significantly down-regulated in the log and late-log phase (Fig. 5) whereas rest TCA genes e.g. *fumA, GBAA_0579, GBAA_4848, acnA, sdhA, sdhB*, and *sdhC*, remain down-regulated throughout the growth. The up-regulation of *yqjl* (2.37) encoding putative hydrolase in the lag phase, suggests that the cells generate NADPH by converting D-gluconate-6-phosphate to D-ribulose-5-phosphate through the pentose phosphate pathway (PPP) pathway. Later this gene was down-regulated in the log phase (−4.15) in the PA expressing culture. Similar to *yqjl*, the nuo and atp operons encoding NADH dehydrogenase and ATP synthase subunits were down-regulated in the PA-expressing culture, indicating a decrease in the energy supply.

### Amino acid biosynthesis

**Figure 6:**
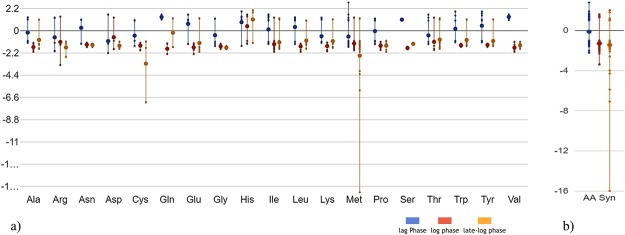
Gene expression profile of amino acid metabolism (**a**) log_2_ fold change of each amino acid gene in PA expressing compared to control, (**b**) Overall gene expression distribution of AA biosynthesis in lag, log, and late-log phases. Large dot represents the average (mean) of all data while small dots represents a data value for an individual gene (using EcoCyc omics dashboard).

Although the cells were growing in the presence of amino acids and short peptides, high regulation of amino acid biosynthesis genes such as *ilvN* (3.02), *tyrA* (1.83), and *GBAA_2958* (1.90) was observed in the lag phase of the PA-expressing cells, probably to fulfil the requirement for precursor amino acids needed for PA expression. As the culture advanced into the log and late-log phase, amino acid biosynthesis slows down in the producing culture compared with the control (Fig. 6). The down-regulation of amino acid transcript limited the supply needed for cellular building block and PA synthesis, pointing towards possible bottleneck where the supply of molecules needed for PA translation is decreased. This was followed by declining rate of recombinant PA expression in log and late-log phase. The slowdown in amino acid biosynthesis with the decreased energy metabolism was associated with the decline in the specific growth rate.

### Transcription and translation

The lag phase data of the PA expressing culture showed up-regulation of 15 tRNA transcripts (>50% of total tRNA transcripts). But later, during the log and the late-log phases more than 95% of the tRNA transcripts were down regulated. Aminoacyl-tRNA synthetase GBAA_1424 (*hisZ*, ATP phosphoribosyltransferase) play a regulatory role and is an essential component of histidine biosynthesis^23^. The PA expressing culture showed up-regulation of *GBAA_1424*, (1.55 in lag, 1.85 in log and 2.52 in late log phases), an indication that this gene is essential for PA biosynthesis. Some RNA polymerase transcripts such as *rpoA, rpoB, rpoC, and rpoE*, were down-regulated throughout PA expression. In addition, a mechanism to keep transcription active was observed by up-regulation of the *rpoZ* transcript in the late-log phase, which facilitates the interaction between alpha and beta subunits of RNA polymerase enzyme assembly.

During the lag and the log phases of the PA expressing culture, there was also up-regulation of *sigL*. This gene encodes the RNA polymerase factor, Sigma 54, that enhances expression of nitrogen assimilation and metabolism genes^24,25^ that reflected in faster growth during lag to log phases. Another study has shown the existence of a catabolite responsive element (CRE) site in *sigL* where transcription factor CcpA (catabolite control protein) binds and blocks mRNA synthesis^26^. Similar regulation was observed in our data where *sigL* expression was higher because its transcriptional repressor *ccpA* was relatively less expressed in the PA producing culture. The general stress transcription factor, *sigB*, was expressed differentially in PA expressing culture. *sigB* together with its regulatory genes, *rsbV* and *rsbW*, in the sigma B operon of *B. anthracis* is organized identical to *B. subtilis* and *B. licheniformis*^27^*. sigB* was found to be induced in *B. subtilis* at the stationary phase while it remains unaffected in *B. anthracis*^28^. In this work *sigB* was up-regualted throughout the growth (lag 2.5, to log 3.0 and latelog 2.7) in the PA expressing strain, together with the regulators of sigma B operon, *rsbV* and *rsbW*. This suggests that *sigB* can also be induced, due to stress features associated with PA expression rather than just at stationary phase. In addition, many other stress related genes were up-regulated in the PA expressing strain such as *htrA, dps, GBAA_1113, GBAA_0326, GBAA_5290* and *GBAA_4875*.

### Protein folding and protein synthesis associated processes

**Figure 7:**
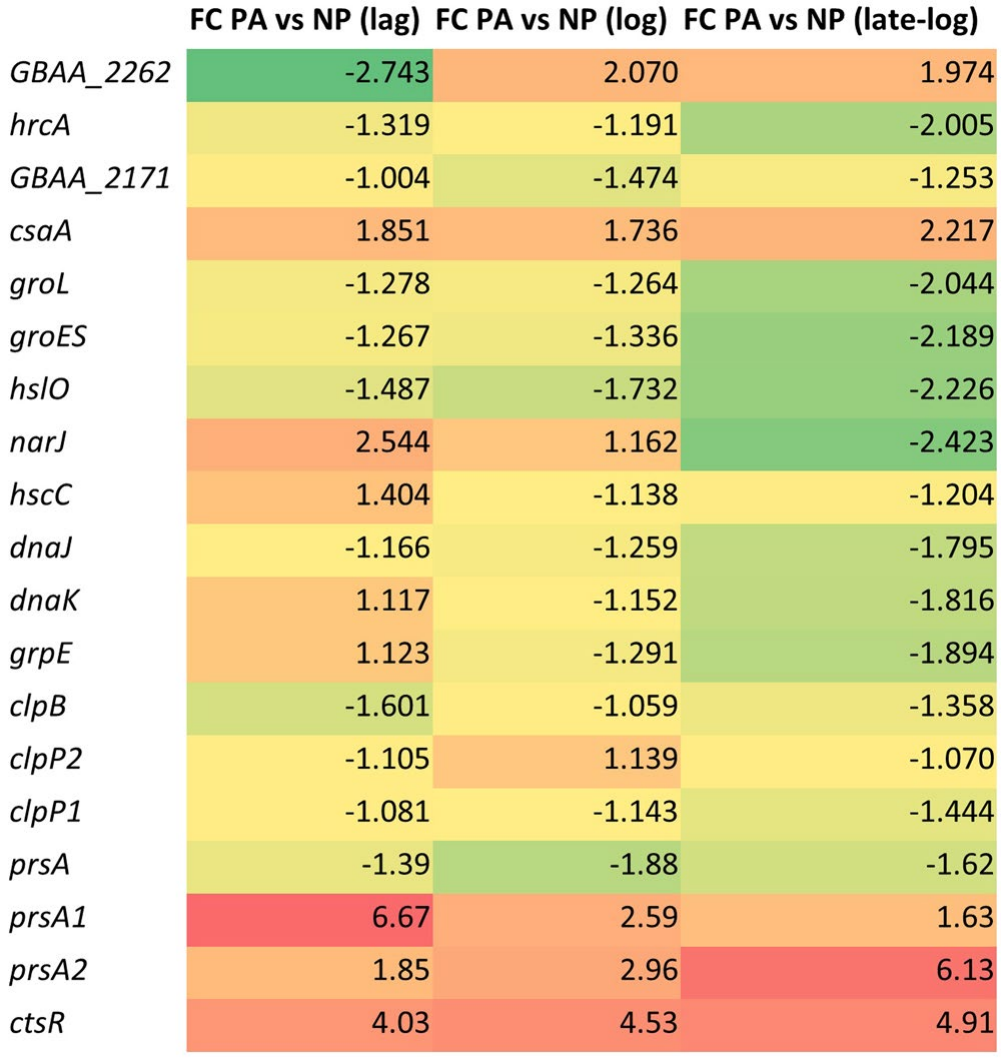
Heatmap of fold-change gene expression values of the heat shock genes, chaperones and DE regulators. FC represents ‘fold change’; PA represents ‘protective antigen producing strain’ and NP represents ‘non-producing strain’.

Protein expression from high copy number plasmids may generate large amounts of nascent polypeptide chains which can outpace the supply of protein folding resources. It was reported, that when recombinant proteins are expressed in Gram positive bacteria, the heat shock proteins (HSPs) were up-regulated^29^ to provide chaperones to assist in protein folding. PA is transported across the cytoplasmic membrane unfolded and later folded by extracellular chaperones. We also observed that *grpE, groEL/ES*, and *dnaK* were down-regulated in PA expressing culture. It is possible that this down-regulation is mediated by HrcA repressor^30,31^. However, since *hrcA* was down-regulated, it cannot be responsible for the repression of *groEL/ES* and *dnaK*. Therefore, if up-regulated HSPs are an indicator of the stress, it is possible that the data presented in this work indicates that PA expression does not impose intracellular folding stress in the BH500 strain. This was strengthened with the finding that the *clp* genes (encoding Clp ATP dependent proteases) that have a role in stress survival^32^, were down-regulated in PA expressing culture. It is possible that the *clp* genes were down-regulated because of the regulatory repression of *ctsR*, stress and heat shock repressor belonging to class III heat shock response in Gram positive bacteria^33^. The *ctsR* gene was up-regulated in lag (4.03), log (4.53) and late-log (4.91) in PA expressing culture (Fig. 7).

### Extracellular secretion

Secretion of expressed proteins is a characteristic of the *Bacillus* species^34,35^, so it is not surprising that *csaA*, a key protein secretion chaperone, was up-regulated in the PA expressing strain throughout the growth. It was reported that *csaA* expression prevented *in-vitro* aggregation of thermally inactivated luciferase^36^ by interaction with secreted precursor proteins^37^. Although expression of *csaA* in *E. coli* was shown to prevent growth and secretion issues^38^ this was not the case in the *B. anthracis*. It is possible that in the *Bacillus*, this gene is responsible for maintaining efficient PA secretion and thereby prevents the impact of protein accumulation on cellular health and growth. Another protein secretion chaperone, PrsA, was reported to play a crucial role in cell viability, folding, and expression of alpha-amylases, and that an increased *prsA* copy number led to a six-fold increase in alpha-amylase secretion in *B. subtilis*^39^. There are three *prsA* homologues in *B. anthracis* as compared to just one copy of *prsA* genes in *B. subtilis*. The three homologues in *B. anthracis* have shown to have a different effect on the rPA yield and it was suggested that it might be because of the their different or overlapping substrate specificities^40^. It is possible that they have completely different functions and selecting the right homologues for manipulation could lead to significant improvement in the rPA yield. In the present study, *prsA1* and *prsA2* were up-regulated while *prsA* remained down-regulated in the PA producing culture. The expression trends of *prsA1* and *prsA2* were opposite; *prsA1* was highly up-regulated in lag-phase (6.6), which later decreased to 1.6-fold in late-log phase, and *prsA2* was less up-regulated (1.8) in the lag-phase, and then increased to 6.1 in the late-log phase (Fig. 7). The higher expression of homologue *prsA2* during higher expression of rPA in the late log phase suggests its key role in increasing production level of properly folded protein, therefore this gene could be a candidate for further improving PA expression.

### Transporter genes

Transporter genes that are responsible for nutrient intake and for product secretion were expressed differentially in the PA-expressing bacteria compared to the control strain (Supplementary Table 2). In the PA expressing bacteria, the heavy metal transporting ATPase *GBAA_0595* involved in maintaining heavy metal homeostatis^41,42^ was up-regulated until the late-log phase (2.6) while the *GBAA_3859* and *GBAA_0410* were down-regulated, indicating reduced cellular health. Glycine betaine/L-proline ABC transporter, permease and substrate-binding protein *GBAA_2280* (1.5) remained up-regulated in the PA expressing strain during lag, log, and late-log phase, while glycine betaine transporter gene *opuD1* (1.1), glycine betaine/L-proline ABC transporter, and ATP-binding protein *proV1* (1.5) were only up-regulated in the late-log phase. The up-regulation of these transport membrane proteins supports the import of osmolytes like glycine betaine and proline from the culture medium, which assist in maintaining proper osmolarity that prevents misfolding or aggregation of expressed proteins^43,44^. Although PA is secreted, it is likely that its increased biosynthesis during late-log phase required more osmolarity adjustment, which was reflected in up-regulation of an additional number of osmolyte transport genes. In addition, the ABC transporter genes which facilitate import of small peptides by carrying out ATP hydrolysis^45,46^ and transfer nutrients from culture broth and support growth of bacteria^47^ were differentially expressed. For example, the ATP binding protein *GBAA_0384*, was up-regulated (>3 fold) in lag, log, and late-log phases of PA expressing culture compared with the control. At the same time, the expression level of amino acid ABC transporter substrate-binding protein *GBAA_0855*, amino acid ABC transporter permease *GBAA_0856*, and amino acid ABC transporter ATP-binding *GBAA_0857* protein, were down regulated −4.37, −6.33, and −8.16 respectively. Also, the complete operon of oligopeptide ABC transporter permease that includes five genes: *GBAA_0231, GBAA_0232, GBAA_0233, GBAA_0234, and GBAA_0235* was down-regulated. The decrease in expression of amino acid and oligopeptide ABC transporters indicates the decline in the culture capability to import nutrients, which directly affects the cellular health and recombinant PA protein expression.

## Conclusion

The effect of protective antigen expression in *Bacillus anthracis* on the profile of the bacterial transcriptome is presented in this work. The expressing strain was compared with strain carrying empty plasmid at all three growth phases. This unique approach eliminated the background noise caused by antibiotics, expression of plasmid genes and natural growth phase transition. By using Venn diagram among the three growth phases of rPA expressing strain comparing it to the non-expressing strain, a set of 24 genes, mostly not well characterized, were found to be differentially expressed in all three growth phases. These genes were not differentially expressed in the control strain, representing their unique connection with expression of rPA (Fig. 3). Log to late-log phase transition was associated with decline in specific growth rate of the expression culture and down-regulation of several biosynthetic pathways. Especially six genes (*GBAA_1162, hemH2, carB, GBAA_2459, sigB, GBAA_4072*) were found to be differentially expressed in the log and late-log phase of rPA expressing culture (Table 2).

**Figure 8:**
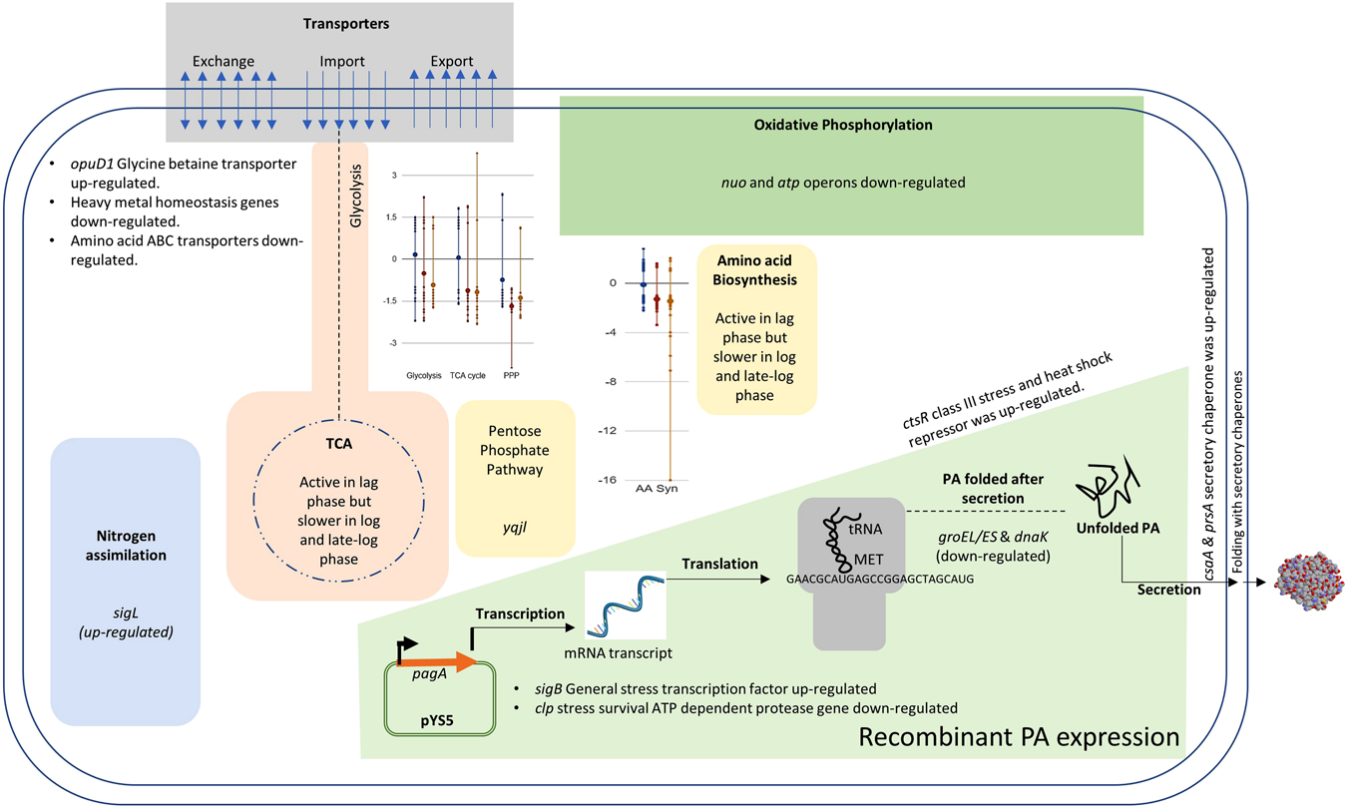
Schematic representation of major pathways significantly affected in the rPA expressing *B. anthracis*.

A schematic of major metabolic pathways and functions affected in *B. anthracis* due to rPA expression are presented in Fig. 8. The upregulation of *sigL* the gene encoding RNA polymerase sigma factor 54 is especially important, it increased the expression of nitrogen assimilation and metabolism genes that likely improve growth rate and rPA expression. Increased expression of the down-regulated *sigL* gene in the late-log phase might support improved growth rate and rPA expression. In addition, up-regulation of the extracellular secretion chaperones *csaA* and *prsA* may increase the rPA export. The *csaA* was shown to be effective in solving secretion issues in *E. coli*^38^, while increased expression of *prsA* lead to improved expression of alpha amylase and rPA in *B. subtilis*^39^ and *B.anthracis*^40^ respectively. This study predicted *prsA* to be an important target which matched with the previous findings^3^. This study also identified that only two out of three *prsA* variants i.e. *prsA1* and *prsA2* might be good candidates for improved rPA expression in *B.anthracis ames* strain.

## Materials and Methods

### Strains and plasmids

*B*. *anthracis* Ames BH500 strain^11^ with the genotype pXO1-, pXO2-, Spo0A- (GBAA_4394), NprB- (0599), TasA- (1298), Cam- (1290), InhA1- (1295), InhA2- (0672), MmpZ- (3159), CysP1- (1995), VpR- (4584), NprC- (2183), S41- (5414) was used in this study. Genetic maps for both plasmids are presented in the Supplementary Fig. S1. The plasmid pYS5 contains original *pagA* promoter from pXO1, including 162 bp of *B. anthracis* DNA upstream of the PA start codon. This promoter comprises both P1 and P2 start transcription sites identified by Koehler *et al*.^48^. The *pagA* and the upstream region were inserted into pUB110 plasmid downstream of kanamycin and truncated bleomycin resistance genes, producing pYS5 plasmid. Both antibiotic resistance genes contain own promoters. Plasmid pSW4 represents pYS5 plasmid with deleted *pagA* gene^49^. The strain was transformed with plasmid pYS5^50^ expresses PA, and the empty parental plasmid pSW4^51^.

### Batch fermentation

Batch growths were performed in Biostat MD fermenter/Bioreactors (B. Braun Biotech International, Germany) in 2.5 liters. Six batch growths, three with BH500 (pYS5) and three with BH500 (pSW4), were performed under identical conditions to achieve biological triplicates for each strain. Modified FA medium containing (per liter) 35 g Soy peptone E110 (Organotechnie, La Courneuve, France), 5 g yeast extract (Difco Laboratories, Detroit, MI, USA), and 100 ml of 10X salt solution, was used. The 10X salt solution consisted of (grams per liter) 60 g Na_2_HPO_4_.7H_2_O, 10 g KH_2_PO_4_, 55 g NaCl, 0.4 g L-tryptophan, 0.4 g L-methionine, 0.05 g thiamine, and 0.25 g uracil and was filter sterilized. The pH of the medium was adjusted to 7.5 with 10% phosphoric acid and 2 N NaOH. Starting cultures were inoculated from frozen stocks and grown for 12–14 hr. Fermenters were inoculated with 3% of the overnight grown culture. The medium in the fermenter was supplemented with 0.2 ml/l of antifoam 289 (Sigma, St. Louis, MO). The growth was conducted at 37 °C and 30% oxygen saturation, which was maintained by varying agitation speed and air flow rate through adaptive control^52^. Culture pH was monitored and controlled only when it started to rise above the set point of 7.5.

### Sample collection

Samples were collected at three different growth phases: lag phase (OD 3), log phase (OD 10), and late-log phase (OD 16). All dilutions and washes to store cells for further analysis were done in PBS containing 1 mM CaCl_2_ and 2 mM MgCl_2_. Each sample was measured after dilution to an 0.2–0.5 OD_600_. Based on these readings, and within 3–5 min, a sample of 3–10 ml was removed and immediately diluted to give >30 ml at a calculated A_600_ = 1.0. This diluted cell suspension was treated differently for different analysis: (i) for RNA-seq: 4.0 ml was added into tubes containing 40 ml RNAprotect (Qiagen, Valencia, Calif.), and the contents were immediately mixed and incubated for 5 min at room temperature. The cells were concentrated by centrifugation at 4000 g, supernatant discarded and the pellet was suspended in 1 ml TRIzol Reagent (GIBCO BRL, Invitrogen), quickly frozen in dry ice and stored at −80 °C; (ii) for protein content determination: 1 ml was pelleted in microfuge tubes, washed with PBS, and the pellets were frozen for BCA assay; *(iii) for determination of plasmid retention, cfu, and purity: two 1 ml samples were diluted to 10^4^- and 10^6^-fold and 100 µl was plated on LB agar plates (no antibiotics), and isolated colonies were streaked to LB agar plates with and without 50 µg/ml kanamycin; (iv) for viability measurement: two 1 ml samples collected at mid-log and late-log time points were assayed with a live/dead dye stain, (v) for PA quantitation using SDS-PAGE: undiluted 1 ml sample was centrifuged and supernatant was collected, filtered through 0.22 µm and stored at −20 °C.

### Plasmid stability test

Time point samples from each run were collected and diluted 10^4^- and 10^6^-fold into PBS and 100 µl from each was plated on LB plates with and without kanamycin (50 µg/ml). Each colony from the non-antibiotic plate was re-streaked on kanamycin plate (50 µg/ml). LB plates were incubated at 37 °C overnight.

### Quantitation of total protein and PA

Total protein in diluted culture samples was determined by BCA assay. Supernatants collected from each time point (fraction ‘v’) were diluted to quantify extracellular PA. Quantification was done by SDS-PAGE (Invitrogen/Novex, Carlsbad, CA) gel analysis, with purified PA as a standard using ImageQuant TL 8.1 software (GE Healthcare).

### Live/dead cell assay

Live/dead Baclight bacterial viability kit (Molecular Probes Europe, Leiden, The Netherlands) was used to determine cell viability.

#### RNA isolation

Sample fraction, frozen in Trizol, was thawed and RNA was extracted by a hot phenol method. The pellets were resuspended in 0.5% SDS, 20 mM NaAcetate, and 10 mM EDTA and extracted twice with hot (60 °C) acid phenol:chloroform (5:1, v/v) followed by two extractions with phenol:chloroform:isoamyl alcohol (25:24:1, v/v). Absolute ethanol was added and the extract was kept at −80 °C for 15 min. After centrifugation at 14,000 g for 15 min, the pellets were washed in 70% ethanol. RNA was airdried and resuspended in ultrapure water (KD medical USA). The quality of each RNA sample was assessed using a Bioanalyzer (Agilent, Santa Clara, CA) and samples with RNA integrity number >8.0 were used for RNA-seq library preparation. RNA from each replicate was extracted at the same time to avoid the batch effect. TURBO™ DNase (Invitrogen/Novex, Carlsbad, CA) was used to remove trace amounts of DNA contamination. The absence of DNA in the samples was confirmed with qPCR of specific genes which showed no amplification up to 25 cycles.

#### Depletion of ribosomal RNA

Enrichment of mRNA was performed by selectively removing ribosomal RNAs using Ribominus transcriptome isolation kit (Invitrogen, Carlsbad, CA, USA). A 10-μg portion of *B. anthracis* total RNA was treated with DNaseI and adsorbed to Locked Nucleic Acid (LNA) probes for 16S and 23S rRNA linked to the magnetic beads which specifically removes 95–98% of rRNA molecules. The enriched RNA pool was concentrated using RiboMinus concentration module (Invitrogen; Thermo Fisher Scientific, Inc.). This RNA was checked for absence of major rRNA peaks in Bioanalyzer 2100 (Agilent Technologies).

#### RNA-seq library preparation

NEBNext® Ultra™ RNA Library Prep Kit (NEB, Ipswich, MA, USA) for Illumina was used. The enriched intact RNA was fragmented to achieve fragment sizes ~200 bp by incubating the reaction mix for 15 min at 94 °C. First strand cDNA synthesis was performed using NEBNext first strand synthesis reaction buffer, random primers, Protoscript II reverse transcriptase in a thermal cycler for 10 min at 25 °C, 15 min at 42 °C and 15 min at 70 °C. To the same reaction mix, second strand synthesis enzyme mix was added and synthesis was performed by incubating the sample at 16 °C for 1 h. The synthesized cDNA strands were purified using a 1:1 ratio of Agencourt AMPure XP beads (Beckman Coulter Genomics, Danvers, MA) and eluted with 60 µl of 0.1X TE Buffer. Later steps involved end repair of extracted cDNA, adapter ligation using blunt/TA ligase and cleavage by USER enzyme, purification with AMPure XP beads and PCR enrichment of cDNA library using different sets of Multiplex oligos for Illumina. PCR enrichment mix was performed in thermocycler for initial denaturation (1 cycle): 98 °C for 30 secs; Denaturation-Annealing/Extension (15 cycles): 98 °C for 10 secs, 65 °C for 75 secs; final extension (1 cycle): 65 °C for 5 min. The enriched library was purified with Agencourt AMPure XP beads (Beckman Coulter, USA), and quality was checked on a Bioanalyzer using Agilent DNA high sensitivity chips.

### Transcriptome sequencing

cDNA library samples for sequencing in Hiseq 2500 (Illumina) were quantitated using PicoGreen assay for dsDNA. 20 ng from nine libraries with different adapter sequences were pooled and run in the single lane of the next generation sequencing chip. Therefore, all 18 samples were processed in two lanes of the Hiseq 2500 single run. This single end sequencing was done to generate 50-bp Illumina sequencing reads.

### RNA-seq data processing

PartekFlow (Partek Inc., St Louis, MO, USA) was used for the bioinformatic analysis. The QA/QC of these unaligned reads were checked to get an insight into the sequencing quality and the average read quality was close to ~40%. The unaligned reads were trimmed to remove low-quality bases from the 3-prime end and checked for quality. These reads were aligned with the reference organism *B. anthracis* Ames ancestor AE017334.2, using Bowtie2 method, which resulted in >97% alignment for each sample. The annotation model file from Ensembl was used to quantify transcriptome where we used the Partek E/M algorithm. PCA was performed to look for the distance between the sample populations, which showed that replicates for a given time point were closer to each other than to replicates of other time points. Before comparing different time point data, normalization was performed to minimize the impact of possible sources of systemic variations, e.g. sequencing depth, gene length, composition, or technical variations. Normalization was performed using the transcripts per million (TPM) method, and then +1 value was added to expression value before taking log_2_. Three biological replicates for each time point sample were used to perform statistical tests to identify differentially expressed genes.

### Pathway and network analysis

Pathway enrichment analysis on the differentially expressed genes was done by integrating *B. anthracis* Ames KEGG pathway database with the Partek Pathway tool in Partek Genomics Suite (Partek Inc., St Louis, MO, USA). The pathway enrichment, as well as its ranking were analyzed based on p-value and fold-change values. EcoCyc database and previous literature was also used to look for gene functions and their possible interconnections. Since many genes in the *B. anthracis* Ames ancestor genome are not annotated, BLAST was also used to find homologous genes and infer function^53^.

### Data submission

The RNA-seq raw and processed data files were deposited in the NCBI Gene Expression Omnibus (GEO) database with the following accession number: GSE108973.

## Electronic supplementary material

Supplementary Information

## Data Availability

RNA-seq data are available in the NCBI Gene Expression Omnibus (GEO) database with the following accession number: GSE108973.
