# Supplementary material for: Effect of over expressing protective antigen on global gene transcription in Bacillus anthracis BH500
Source: Sci Rep. 2018 Oct 31;8:16108. doi: 10.1038/s41598-018-34196-y (PMC6208434; doi:10.1038/s41598-018-34196-y)
Supplement: Supplementary file 1 — Supplementary Information [file 41598_2018_34196_MOESM1_ESM.pdf]

Supplementary Information:

Article in *Scientific Reports*

Effect of over expressing protective antigen on global gene transcription in *Bacillus anthracis* BH500.

Ashish Kumar Sharma[1], Stephen H. Leppla[2], Andrei P. Pomerantsev[2], and Joseph Shiloach[1*]

[1]Biotechnology Core Laboratory, National Institute of Diabetes and Digestives and Kidney Diseases (NIDDK) NIH

[2]Microbial Pathogenesis Section, National Institute of Allergy and Infectious diseases (NIAID), NIH

[*]Correspondence to JosephS@niddk.nih.gov

Supplementary Figure S1. Genetic maps of pYS5 and pSW4 plasmids. pSW4 is similar to pYS5 with *pagA* deleted (Pomerantsev et al., 2003).

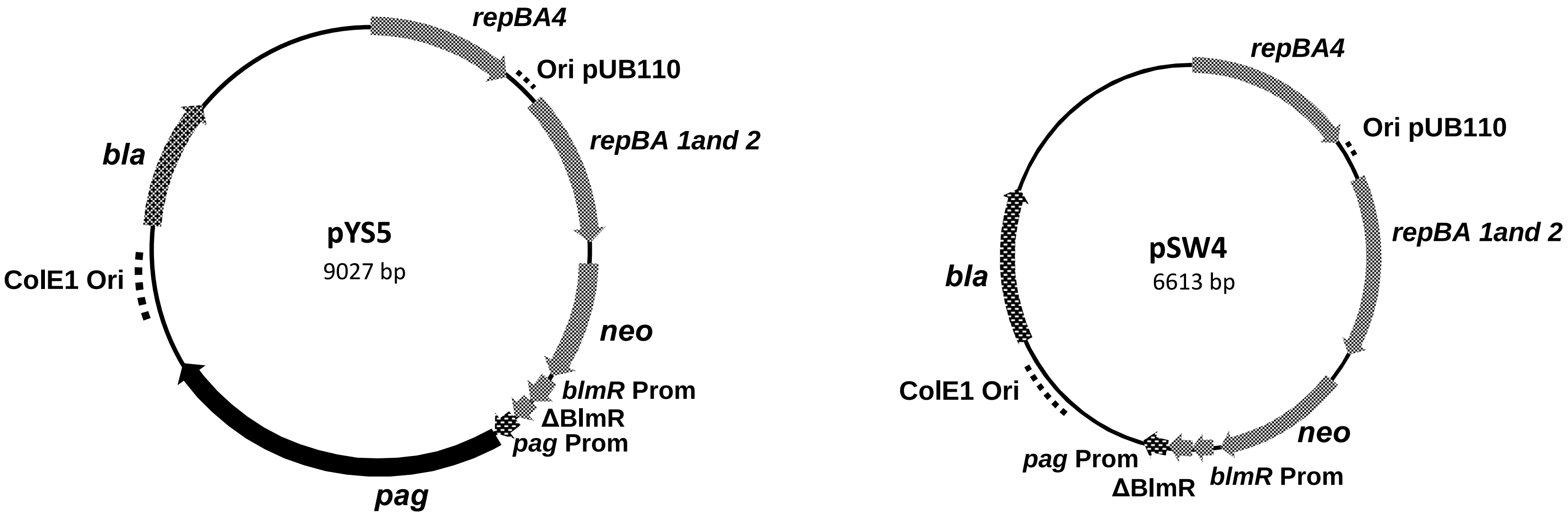

Supplementary Table 1. Pathway enrichment outcome along with their enrichment scores in lag, log and late-log phases. (Differentially expressed at lag, log and late-log phase of PA producing vs non-producing cultures were used for pathway enrichment analysis.)

Lag Phase Pathway Enrichment						
**Pathway Name**	**Enrichment Score**	**Enrichment p-value**	**% genes in pathway that are present**	**# genes in list, in pathway**	**# genes not in list, in pathway**	**KEGG Pathway ID**
Pyrimidine metabolism	4.15	0.02	7	4	54	54
Alanine, aspartate and glutamate metabolism	3.95	0.02	9	3	31	113
beta-Lactam resistance	3.01	0.05	10	2	19	14
Flagellar assembly	2.84	0.06	9	2	21	59
NOD-like receptor signaling pathway	2.69	0.07	25	1	3	106
Cyanoamino acid metabolism	2.31	0.10	17	1	5	103
Glycolysis / Gluconeogenesis	1.43	0.24	4	2	52	70
Sulfur relay system	1.26	0.28	5	1	18	67
Nicotinate and nicotinamide metabolism	1.26	0.28	5	1	18	83
Tryptophan metabolism	1.21	0.30	5	1	19	31
Phenylalanine, tyrosine and tryptophan biosynthesis	1.14	0.32	5	1	21	26
Carbon metabolism	1.08	0.34	3	3	116	41
Glycerophospholipid metabolism	1.06	0.34	4	1	23	101
Pentose phosphate pathway	0.97	0.38	4	1	26	16
Folate biosynthesis	0.94	0.39	4	1	27	64
Citrate cycle (TCA cycle)	0.91	0.40	3	1	28	23
Glyoxylate and dicarboxylate metabolism	0.84	0.43	3	1	31	88
Metabolic pathways	0.78	0.46	2	12	638	97
ABC transporters	0.77	0.46	2	3	141	6

Biosynthesis of secondary metabolites	0.73	0.48	2	6	311	40
Quorum sensing	0.63	0.53	2	2	99	49
Biosynthesis of antibiotics	0.55	0.58	2	4	225	45
Pyruvate metabolism	0.47	0.63	2	1	54	82
Aminoacyl-tRNA biosynthesis	0.39	0.68	2	2	128	85
Biosynthesis of amino acids	0.34	0.71	1	2	137	36
Two-component system	0.18	0.84	1	1	99	87
Microbial metabolism in diverse environments	0.18	0.84	1	2	177	20
Log Phase Pathway Enrichment						
**Pathway Name**	**Enrichment Score**	**Enrichment p-value**	**% genes in pathway that are present**	**# genes in list, in pathway**	**# genes not in list, in pathway**	**Pathway ID**
Pyrimidine metabolism	5.28	0.01	9	5	53	54
Flagellar assembly	4.61	0.01	13	3	20	59
Porphyrin and chlorophyll metabolism	2.91	0.05	11	2	17	57
Pyruvate metabolism	1.18	0.31	4	2	53	82
Biotin metabolism	1.17	0.31	6	1	17	62
Lysine biosynthesis	1.13	0.32	5	1	18	28
Nicotinate and nicotinamide metabolism	1.13	0.32	5	1	18	83
ABC transporters	1.11	0.33	3	4	140	6
Tryptophan metabolism	1.09	0.34	5	1	19	31
Bacterial chemotaxis	0.97	0.38	4	1	22	100
Fatty acid biosynthesis	0.88	0.41	4	1	25	79
Pentose phosphate pathway	0.85	0.43	4	1	26	16
Carbon metabolism	0.83	0.44	3	3	116	41
Citrate cycle (TCA cycle)	0.80	0.45	3	1	28	23
Glyoxylate and dicarboxylate metabolism	0.73	0.48	3	1	31	88
Fatty acid metabolism	0.73	0.48	3	1	31	10
Alanine, aspartate and glutamate metabolism	0.68	0.50	3	1	33	113

Starch and sucrose metabolism	0.63	0.54	3	1	36	15
Two-component system	0.49	0.61	2	2	98	87
Quorum sensing	0.48	0.62	2	2	99	49
Glycolysis / Gluconeogenesis	0.39	0.68	2	1	53	70
Aminoacyl-tRNA biosynthesis	0.28	0.76	2	2	128	85
Metabolic pathways	0.17	0.84	2	11	639	97
Ribosome	0.17	0.85	1	1	88	86
Biosynthesis of antibiotics	0.14	0.87	1	3	226	45
Microbial metabolism in diverse environments	0.11	0.90	1	2	177	20
Biosynthesis of secondary metabolites	0.09	0.91	1	4	313	40
Biosynthesis of amino acids	0.05	0.95	1	1	138	36
Late-Log Phase Pathway Enrichment						
**Pathway Name**	**Enrichment Score**	**Enrichment p-value**	**% genes in pathway that are present**	**# genes in list, in pathway**	**# genes not in list, in pathway**	**Pathway ID**
Oxidative phosphorylation	12.71	0.00	29	13	32	48
Cysteine and methionine metabolism	8.35	0.00	21	12	45	4
Metabolic pathways	7.29	0.00	9	59	591	97
Citrate cycle (TCA cycle)	6.09	0.00	24	7	22	23
Tryptophan metabolism	4.77	0.01	25	5	15	31
Carbon metabolism	3.76	0.02	12	14	105	41
Sulfur metabolism	3.57	0.03	22	4	14	37
Quorum sensing	3.42	0.03	12	12	89	49
Taurine and hypotaurine metabolism	2.88	0.06	33	2	4	111
Glyoxylate and dicarboxylate metabolism	2.84	0.06	16	5	27	88
Seleno compound metabolism	2.60	0.07	20	3	12	32
Degradation of aromatic compounds	2.14	0.12	22	2	7	18
Biosynthesis of amino acids	2.04	0.13	9	13	126	36
Pyruvate metabolism	1.84	0.16	11	6	49	82

beta-Lactam resistance	1.82	0.16	14	3	18	14
Chlorocyclohexane and chlorobenzene degradation	1.67	0.19	33	1	2	102
Pyrimidine metabolism	1.67	0.19	10	6	52	54
Microbial metabolism in diverse environments	1.56	0.21	8	15	164	20
Pentose phosphate pathway	1.31	0.27	11	3	24	16
Biosynthesis of secondary metabolites	1.27	0.28	8	24	293	40
Vitamin B6 metabolism	1.22	0.29	20	1	4	34
Styrene degradation	1.22	0.29	20	1	4	24
Glycolysis / Gluconeogenesis	1.22	0.29	9	5	49	70
Naphthalene degradation	1.07	0.34	17	1	5	13
Biosynthesis of antibiotics	1.01	0.36	7	17	212	45
Porphyrin and chlorophyll metabolism	1.00	0.37	11	2	17	57
Nicotinate and nicotinamide metabolism	1.00	0.37	11	2	17	83
Sulfur relay system	1.00	0.37	11	2	17	67
ABC transporters	0.99	0.37	8	11	133	6
D-Alanine metabolism	0.95	0.39	14	1	6	8
Histidine metabolism	0.93	0.39	10	2	18	3
Alanine, aspartate and glutamate metabolism	0.91	0.40	9	3	31	113
Thiamine metabolism	0.87	0.42	10	2	19	39
Glycerophospholipid metabolism	0.72	0.49	8	2	22	101
Benzoate degradation	0.69	0.50	10	1	9	109
C5-Branched dibasic acid metabolism	0.69	0.50	10	1	9	53
Chloroalkane and chloroalkene degradation	0.62	0.54	9	1	10	50
Butanoate metabolism	0.56	0.57	7	2	26	19
Methane metabolism	0.56	0.57	7	2	26	30
Tyrosine metabolism	0.47	0.62	7	1	13	65
Lysine degradation	0.47	0.62	7	1	13	80
Phenylalanine metabolism	0.47	0.62	7	1	13	93
Propanoate metabolism	0.41	0.66	6	2	31	11
Glycerolipid metabolism	0.40	0.67	6	1	15	33

Fructose and mannose metabolism	0.40	0.67	6	1	15	71
Arginine and proline metabolism	0.39	0.68	6	2	32	61
Arginine biosynthesis	0.33	0.72	6	1	17	104
RNA degradation	0.33	0.72	6	1	17	96
Phosphotransferase system (PTS)	0.28	0.75	5	1	19	2
Fatty acid degradation	0.28	0.75	5	1	19	94
Glycine, serine and threonine metabolism	0.20	0.82	4	2	43	89
Purine metabolism	0.17	0.84	4	3	64	66
Ribosome	0.15	0.86	4	4	85	86
2-Oxocarboxylic acid metabolism	0.13	0.88	3	1	29	81
Starch and sucrose metabolism	0.08	0.93	3	1	36	15
Aminoacyl-tRNA biosynthesis	0.00	1.00	2	2	128	85

Supplementary Table 2. Log_2 fold change values of differentially expressed genes related to transport in lag, log and late-log phases in PA expressing vs non-expressing control culture.

		**Fold Change in given growth phase**		
**Gene ID**	**Description**	**Lag**	**Log**	**Latelog**
GBAA_0384	ABC transporter, ATP-binding protein	3.495	3.784	3.459
GBAA_1945	transport ATP-binding protein CydC	4.544	1.220	2.945
GBAA_4669	ABC transporter, ATP-binding protein	4.696	2.503	2.752
GBAA_2041	putative oligopeptide ABC transporter, oligopeptide-binding protein	2.485	2.450	2.607
GBAA_5219	putative ABC transporter, substrate-binding protein	1.557	2.175	2.561
GBAA_0595	heavy metal-transporting ATPase	1.362	2.086	2.548
GBAA_4997	ABC transporter, ATP-binding protein	1.228	2.033	2.158
GBAA_4996	putative ABC transporter, permease protein	1.517	1.855	2.014

kdpA	potassium-transporting ATPase, A subunit	-1.242	1.849	1.949
GBAA_4492	putative phosphate transport system regulatory protein PhoU	-1.152	-1.088	1.839
GBAA_5510	techoic acid ABC transporter, ATP-binding protein	3.592	2.819	1.826
GBAA_0262	ABC transporter, ATP-binding protein	2.396	2.376	1.757
GBAA_4668	ABC transporter, permease protein	7.843	3.349	1.606
GBAA_2164	ABC transporter, ATP-binding protein	3.781	2.223	1.591
GBAA_0351	iron compound ABC transporter, iron compound-binding protein	2.216	2.325	1.556
proV1	glycine betaine/L-proline ABC transporter, ATP-binding protein	1.199	-1.185	1.520
GBAA_2280	glycine betaine/L-proline ABC transporter, permease and substrate-binding protein	1.972	1.136	1.508
GBAA_1375	putative ABC transporter, permease protein	1.529	-1.753	1.505
pstA	phosphate ABC transporter, permease protein	-1.118	-1.127	1.503
GBAA_1197	oligopeptide ABC transporter, oligopeptide-binding protein	3.081	1.613	1.475
GBAA_5309	major facilitator family transporter	1.904	1.269	1.431
GBAA_1321	formate/nitrite transporter family protein	1.384	3.156	1.402
GBAA_0228	ABC transporter, ATP-binding protein	1.071	1.274	1.391
phoX	phosphate ABC transporter, phosphate-binding protein	-1.150	1.173	1.389
GBAA_3645	putative oligopeptide ABC transporter, oligopeptide-binding protein	11.419	3.246	1.366
GBAA_2050	magnesium transporter, CorA family	-1.029	-1.028	1.347
pstC	phosphate ABC transporter, permease protein	-1.360	1.029	1.305
GBAA_2228	ABC transporter, ATP-binding protein	-1.378	-1.596	1.279
GBAA_2877	drug resistance transporter, EmrB/QacA family	1.067	1.556	1.277
GBAA_5298	nucleoside transporter, NupC family	-1.348	1.514	1.272
GBAA_0405	cation-transporting ATPase, E1-E2 family	2.181	5.103	1.216
GBAA_3926	sugar ABC transporter, ATP-binding protein	1.862	-1.177	1.191
GBAA_0656	oligopeptide ABC transporter, oligopeptide-binding protein	5.236	1.584	1.171
pstB	phosphate ABC transporter, ATP-binding protein	-1.169	1.085	1.167
GBAA_4766	iron compound ABC transporter, iron compound-binding protein	5.611	1.334	1.151
GBAA_3387	ABC transporter, ATP-binding protein	1.003	1.002	1.150
GBAA_0416	transporter, EamA family	1.087	-1.171	1.145
GBAA_2390	putative ABC transporter, permease protein	2.686	2.586	1.138

modB	molybdenum ABC transporter, permease protein	1.708	1.766	1.126
GBAA_4667	ABC transporter, permease protein	2.975	2.014	1.113
mntA	manganese ABC transporter, manganese-binding protein	1.798	1.032	1.095
GBAA_2896	putative transporter	-1.614	-2.211	1.094
opuD1	glycine betaine transporter	1.144	-1.702	1.087
corA	magnesium and cobalt transport protein CorA	-1.123	-1.640	1.064
GBAA_5085	efflux ABC transporter, ATP-binding protein	-1.102	-1.516	1.052
GBAA_5066	probable proton-coupled thiamine transporter YuaJ	-1.088	-1.508	1.036
GBAA_1374	ABC transporter, ATP-binding protein	1.497	-1.540	1.034
GBAA_2531	ABC transporter, ATP-binding protein	1.353	-1.261	-1.056
GBAA_1191	putative oligopeptide ABC transporter, oligopeptide-binding protein	1.852	-1.015	-1.062
GBAA_2444	ABC transporter, permease/ATP-binding protein	1.376	1.185	-1.062
kdpC	potassium-transporting ATPase, C subunit	-1.535	1.164	-1.090
GBAA_2443	ABC transporter, permease/ATP-binding protein	1.200	-1.012	-1.093
GBAA_0785	Na/Pi-cotransporter family protein	-2.515	-1.839	-1.102
GBAA_0829	transporter, EamA family	1.579	-1.765	-1.122
GBAA_3190	metal ABC transporter, permease protein	1.529	-1.100	-1.128
GBAA_0368	amino acid ABC transporter, ATP-binding protein	1.125	1.427	-1.137
GBAA_3741	ABC transporter, permease/ATP-binding protein	-1.030	-2.207	-1.147
GBAA_2603	ABC transporter, ATP-binding protein	1.106	-1.218	-1.157
kdpB	potassium-transporting ATPase, B subunit	-1.677	1.802	-1.188
GBAA_3191	metal ABC transporter, ATP-binding protein	1.448	-1.143	-1.201
GBAA_5091	ABC transporter, ATP-binding protein	-1.087	1.284	-1.201
GBAA_4505	putative cation ABC transporter, ATP-binding protein	-1.497	-2.463	-1.232
GBAA_5220	putative ABC transporter, substrate-binding protein	1.104	-1.071	-1.239
GBAA_0715	putative phosphate ABC transporter, phosphate-binding protein	1.374	1.210	-1.247
GBAA_5497	efflux transporter, RND family, MFP subunit	1.729	1.415	-1.248
GBAA_1302	putative transporter	1.374	1.077	-1.259
ecsA	ABC transporter, ATP-binding protein EcsA	-1.110	-1.502	-1.269
ftsE	cell division ABC transporter, ATP-binding protein FtsE	-1.211	-2.089	-1.273

GBAA_0533	putative ABC transporter, permease protein	-1.429	-1.928	-1.278
GBAA_3518	drug resistance transporter, Bcr/CflA family	-1.439	-1.715	-1.279
GBAA_5686	transporter, AcrB/AcrD/AcrF family	1.660	1.423	-1.280
GBAA_2948	ABC transporter, ATP-binding protein	-1.142	-1.681	-1.286
GBAA_3447	major facilitator family transporter	1.006	-1.245	-1.297
GBAA_0618	iron compound ABC transporter, ATP-binding protein	-1.109	-1.708	-1.300
GBAA_0200	putative transporter	-1.073	-1.101	-1.327
GBAA_0367	putative amino acid ABC transporter, amino acid-binding protein	1.832	1.182	-1.332
GBAA_5698	putative transporter	-1.567	1.531	-1.332
GBAA_0798	ABC transporter, ATP-binding protein	2.038	1.331	-1.336
GBAA_3020	major facilitator family transporter	1.382	-1.236	-1.357
potD	spermidine/putrescine ABC transporter, spermidine/putrescine-binding protein	-1.166	-1.493	-1.365
GBAA_3424	transporter, EamA family	-1.162	-1.629	-1.365
GBAA_3345	major facilitator family transporter	-1.471	-1.611	-1.371
GBAA_1376	ABC transporter, ATP-binding protein	-1.213	-1.438	-1.385
GBAA_3740	ABC transporter, permease/ATP-binding protein	1.200	-2.070	-1.393
potA	spermidine/putrescine ABC transporter, ATP-binding protein	-1.061	-2.276	-1.401
GBAA_5084	efflux ABC transporter, permease protein	-1.210	-1.530	-1.402
GBAA_3653	ABC transporter, ATP-binding protein	-1.683	-1.575	-1.415
GBAA_1471	phosphate transporter family protein	-1.492	-2.041	-1.421
GBAA_0852	ABC transporter, permease/ATP-binding protein	-1.064	-1.541	-1.428
GBAA_5330	iron compound ABC transporter, iron compound-binding protein	-1.859	-2.104	-1.453
GBAA_1448	putative sodium-dependent transporter	-1.369	-1.757	-1.456
brnQ5	branched-chain amino acid transport system II carrier protein	1.355	1.087	-1.484
GBAA_1126	transporter, EamA family	-1.082	-1.472	-1.486
GBAA_1605	putative cation transporter	1.074	-1.031	-1.499
GBAA_0657	oligopeptide ABC transporter, permease protein	3.515	1.072	-1.500
GBAA_5678	ABC transporter, ATP-binding protein	-1.381	-1.908	-1.500
GBAA_2216	putative sodium-dependent transporter	-1.002	-1.603	-1.504
GBAA_1622	transporter, EamA family	1.096	-1.329	-1.505

GBAA_0797	putative ABC transporter, permease protein	1.119	1.207	-1.521
GBAA_5439	chromate ion transporter	-1.392	-1.351	-1.524
GBAA_5163	putative transporter	-1.060	-1.568	-1.532
GBAA_4299	ABC transporter, ATP-binding protein	1.076	-1.462	-1.535
feoB	ferrous iron transport protein B	1.044	-1.780	-1.537
GBAA_5327	iron compound ABC transporter, ATP-binding protein	-1.572	-1.740	-1.550
GBAA_0284	putative bacitracin ABC transporter, permease protein	-1.073	-1.051	-1.550
malD	maltosaccharide ABC transporter, permease protein	1.427	-1.786	-1.551
GBAA_1377	putative ABC transporter, permease protein	-1.536	-1.396	-1.558
GBAA_5221	ABC transporter, permease protein	-1.001	-1.206	-1.560
GBAA_5495	ABC transporter, permease protein	1.368	1.251	-1.566
GBAA_1819	putative transporter	-1.249	-1.527	-1.568
GBAA_0616	iron compound ABC transporter, permease protein	-1.143	-1.869	-1.577
GBAA_4229	putative maltosaccharide ABC transporter, maltosaccharide-binding protein	1.589	-1.642	-1.585
GBAA_0285	putative bacitracin ABC transporter, ATP-binding protein	1.426	-1.086	-1.587
GBAA_0381	putative ABC transporter, permease protein	-1.536	-1.820	-1.592
GBAA_0195	putative oligopeptide ABC transporter, oligopeptide-binding protein	-1.676	-1.134	-1.596
GBAA_5328	iron compound ABC transporter, permease protein	-1.672	-1.848	-1.609
GBAA_3364	ABC transporter, ATP-binding protein	-1.565	-1.466	-1.611
ecsB	ABC transporter, permease protein EscB	-1.277	-1.568	-1.615
GBAA_0839	putative ABC transporter, substrate-binding protein	1.752	-1.488	-1.616
ftsX	cell division ABC transporter, permease protein FtsX	-1.249	-1.804	-1.620
GBAA_0244	major facilitator family transporter	-1.174	-2.024	-1.633
GBAA_0349	iron compound ABC transporter, permease protein	-1.267	-1.528	-1.636
brnQ2	branched-chain amino acid transport system II carrier protein	1.137	-1.629	-1.639
GBAA_0175	putative ABC transporter, substrate-binding protein	1.670	1.017	-1.640
GBAA_4812	drug resistance transporter, EmrB/QacA family	1.329	-1.082	-1.650
GBAA_5511	putative techoic acid ABC transporter, efflux permease	1.099	1.147	-1.651
GBAA_3574	bile acid transporter family protein	1.301	-1.399	-1.656
GBAA_5496	ABC transporter, ATP-binding protein	1.388	1.277	-1.662

rbsB	ribose ABC transporter, ribose-binding protein	1.246	-1.222	-1.697
potC	spermidine/putrescine ABC transporter, permease protein	-1.263	-1.901	-1.705
GBAA_0615	iron compound ABC transporter, iron compound-binding protein	-1.166	-2.228	-1.707
GBAA_0534	putative ABC transporter, permease protein	-1.865	-1.820	-1.730
GBAA_1195	oligopeptide ABC transporter, ATP-binding protein	-1.123	-1.415	-1.737
GBAA_5329	iron compound ABC transporter, permease protein	-1.489	-1.885	-1.743
GBAA_5629	iron compound ABC transporter, ATP-binding protein	-1.312	-2.044	-1.757
GBAA_5650	ABC transporter, ATP-binding protein	-1.358	-1.741	-1.757
GBAA_1038	drug resistance transporter, EmrB/QacA family	-1.314	-1.608	-1.758
GBAA_0181	drug resistance transporter, Bcr/CflA family	-1.574	-1.919	-1.803
GBAA_1858	major facilitator family transporter	-2.348	-2.227	-1.808
GBAA_5222	ABC transporter, ATP-binding protein	-1.237	-1.330	-1.821
GBAA_5024	transporter, EamA family	-1.117	-1.201	-1.847
GBAA_1333	sodium transporter family protein	-1.143	-1.856	-1.857
GBAA_3223	drug resistance transporter, EmrB/QacA family	-1.364	-1.800	-1.867
GBAA_5649	ABC transporter, permease protein	-1.587	-1.482	-1.870
GBAA_5175	ABC transporter, ATP-binding protein	-1.445	-1.471	-1.871
GBAA_3157	putative transporter	-1.356	-1.917	-1.877
GBAA_0410	heavy metal-transporting ATPase	-1.048	-1.182	-1.895
GBAA_4767	iron compound ABC transporter, permease protein	1.212	-1.243	-1.901
GBAA_0800	putative ABC transporter, permease protein	-1.629	-2.261	-1.919
GBAA_1014	putative transporter	-3.220	-1.741	-1.923
GBAA_0801	putative ABC transporter, permease protein	-1.287	-1.303	-1.924
GBAA_0139	ABC transporter, ATP-binding protein	-1.217	-1.565	-1.927
GBAA_3342	major facilitator family transporter	-1.364	-1.801	-1.945
GBAA_0944	drug resistance transporter, EmrB/QacA family	-1.408	-2.202	-1.952
GBAA_0140	ABC transporter, ATP-binding protein	-1.296	-1.420	-1.984
GBAA_1893	nucleoside transporter NupC	1.120	1.208	-1.990
GBAA_0378	anaerobic C4-dicarboxylate membrane transporter	-1.005	-1.124	-1.995
brnQ4	branched-chain amino acid transport system II carrier protein	-1.183	-1.440	-2.031

GBAA_0173	ABC transporter, permease protein	-1.107	-1.531	-2.031
potB	spermidine/putrescine ABC transporter, permease protein	-1.255	-2.026	-2.036
GBAA_1194	oligopeptide ABC transporter, ATP-binding protein	-1.042	-1.535	-2.036
narK	nitrate transporter	1.056	-1.320	-2.039
GBAA_0617	iron compound ABC transporter, permease protein	-1.218	-2.108	-2.048
GBAA_4961	drug resistance transporter, EmrB/QacA family	-1.812	-3.041	-2.051
GBAA_2130	ABC transporter, ATP-binding protein	-1.532	-1.354	-2.052
GBAA_2991	nucleoside transporter, NupC family	-1.285	-1.366	-2.056
malC	maltosaccharide ABC transporter, permease protein	-1.053	-1.878	-2.065
GBAA_4504	putative cation ABC transporter, permease protein	-1.509	-2.372	-2.096
rbsC	ribose ABC transporter, permease protein	1.215	-1.250	-2.109
GBAA_0382	putative ABC transporter, substrate-binding protein	-1.125	-1.559	-2.159
GBAA_0174	ABC transporter, ATP-binding protein	1.278	-1.587	-2.162
GBAA_0787	major facilitator family transporter	-1.357	-2.283	-2.174
GBAA_0528	ABC transporter, permease/ATP-binding protein	-1.560	-1.810	-2.175
GBAA_0643	amino acid ABC transporter, permease protein	-1.074	-1.275	-2.191
GBAA_0716	putative phosphate ABC transporter, permease protein	-1.213	-1.623	-2.196
GBAA_0658	oligopeptide ABC transporter, permease protein	2.608	-1.104	-2.226
GBAA_0717	putative phosphate ABC transporter, permease protein	-1.585	-1.797	-2.261
GBAA_0383	ABC transporter, ATP-binding protein	-1.029	-1.502	-2.273
GBAA_5411	ABC transporter, permease/ATP-binding protein	-1.670	-2.088	-2.276
brnQ3	branched-chain amino acid transport system II carrier protein	1.100	-1.192	-2.310
GBAA_1880	transport protein, NRAMP family	-1.316	-1.430	-2.321
GBAA_0532	ABC transporter, ATP-binding protein	-1.596	-2.021	-2.327
GBAA_0141	cobalt transport protein	-1.364	-1.525	-2.349
glpT	glycerol-3-phosphate transporter	-1.711	-2.312	-2.359
rbsD	ribose ABC transporter protein	1.375	-1.103	-2.388
rbsA	ribose ABC transporter, ATP-binding protein	1.647	-1.168	-2.428
GBAA_3451	serine/threonine transporter family protein	-1.600	-1.828	-2.437
GBAA_5668	major facilitator family transporter	-2.436	-2.044	-2.443

GBAA_0660	oligopeptide ABC transporter, ATP-binding protein	1.811	-1.107	-2.460
GBAA_1193	oligopeptide ABC transporter, permease protein	1.009	-1.636	-2.466
GBAA_1759	transporter, EamA family	-1.664	-1.698	-2.502
GBAA_1192	oligopeptide ABC transporter, permease protein	1.079	-1.825	-2.525
GBAA_0659	oligopeptide ABC transporter, ATP-binding protein	1.978	-1.281	-2.539
GBAA_5630	iron compound ABC transporter, permease protein	-1.228	-3.410	-2.585
brnQ6	branched-chain amino acid transport system II carrier protein	-1.040	-2.515	-2.629
GBAA_5742	ABC transporter, permease protein	1.010	-1.809	-2.694
GBAA_0350	iron compound ABC transporter, permease protein	-1.275	-1.289	-2.768
GBAA_4232	sugar ABC transporter, ATP-binding protein	-1.671	-1.685	-2.882
GBAA_5743	putative amino acid ABC transporter, permease protein	-1.461	-1.505	-3.027
GBAA_3369	putative transporter	-1.598	-2.262	-3.639
GBAA_4216	drug resistance transporter, EmrB/QacA family	-1.332	-1.888	-3.850
GBAA_0908	oligopeptide ABC transporter, oligopeptide-binding protein	1.737	1.756	-4.272
GBAA_5475	nucleoside transporter, NupC family	-2.672	-2.636	-4.488
GBAA_3267	major facilitator family transporter	-1.223	-1.076	-4.625
GBAA_3859	heavy metal-transporting ATPase	-4.033	-4.769	-4.734
GBAA_5260	major facilitator family transporter	-3.501	-7.128	-4.737
GBAA_0855	amino acid ABC transporter, amino acid-binding protein	-1.101	-1.961	-5.033
opuD2	glycine betaine transporter	1.054	-1.675	-5.137
GBAA_0313	ABC transporter, permease protein	-1.097	-1.461	-5.393
GBAA_0390	major facilitator family transporter	-1.699	-1.872	-6.619
GBAA_0912	oligopeptide ABC transporter, ATP-binding protein	1.281	1.096	-6.636
GBAA_0856	amino acid ABC transporter, permease protein	-1.311	-2.254	-7.738
GBAA_0909	oligopeptide ABC transporter, permease protein	1.063	-1.047	-8.185
GBAA_0857	amino acid ABC transporter, ATP-binding protein	-1.493	-2.106	-9.783
GBAA_0314	putative ABC transporter, substrate-binding protein	-1.231	-1.499	-9.832
GBAA_0910	oligopeptide ABC transporter, permease protein	-1.059	-1.068	-11.488
GBAA_0231	putative oligopeptide ABC transporter, oligopeptide-binding protein	1.583	-1.046	-13.965
GBAA_0235	oligopeptide ABC transporter, ATP-binding protein	-1.549	-1.301	-14.847

GBAA_0232	oligopeptide ABC transporter, permease protein	-1.257	-1.807	-15.083
GBAA_0233	oligopeptide ABC transporter, permease protein	-1.573	-1.686	-15.422
GBAA_3492	ABC transporter, efflux permease protein	-10.701	-17.433	-15.924
GBAA_3493	ABC transporter, ATP-binding protein	-14.725	-19.743	-16.970
GBAA_0234	oligopeptide ABC transporter, ATP-binding protein	-1.526	-1.414	-19.292
